# NADPH Oxidase 1 Is Associated with Altered Host Survival and T Cell Phenotypes after Influenza A Virus Infection in Mice

**DOI:** 10.1371/journal.pone.0149864

**Published:** 2016-02-24

**Authors:** Amelia R. Hofstetter, Juan A. De La Cruz, Weiping Cao, Jenish Patel, Jessica A. Belser, James McCoy, Justine S. Liepkalns, Samuel Amoah, Guangjie Cheng, Priya Ranjan, Becky A. Diebold, Wun-Ju Shieh, Sherif Zaki, Jacqueline M. Katz, Suryaprakash Sambhara, J. David Lambeth, Shivaprakash Gangappa

**Affiliations:** 1 Influenza Division, Centers for Disease Control and Prevention, Atlanta, Georgia, United States of America; 2 Department of Pathology and Laboratory Medicine, Emory University, Atlanta, Georgia, United States of America; 3 Infectious Disease Pathology Branch, Centers for Disease Control and Prevention, Atlanta, Georgia, United States of America; University of Missouri-Columbia, UNITED STATES

## Abstract

The role of the reactive oxygen species-producing NADPH oxidase family of enzymes in the pathology of influenza A virus infection remains enigmatic. Previous reports implicated NADPH oxidase 2 in influenza A virus-induced inflammation. In contrast, NADPH oxidase 1 (Nox1) was reported to decrease inflammation in mice within 7 days post-influenza A virus infection. However, the effect of NADPH oxidase 1 on lethality and adaptive immunity after influenza A virus challenge has not been explored. Here we report improved survival and decreased morbidity in mice with catalytically inactive NADPH oxidase 1 (Nox1*^/Y^) compared with controls after challenge with A/PR/8/34 influenza A virus. While changes in lung inflammation were not obvious between Nox1*^/Y^ and control mice, we observed alterations in the T cell response to influenza A virus by day 15 post-infection, including increased interleukin-7 receptor-expressing virus-specific CD8^+^ T cells in lungs and draining lymph nodes of Nox1*^/Y^, and increased cytokine-producing T cells in lungs and spleen. Furthermore, a greater percentage of conventional and interstitial dendritic cells from Nox1*^/Y^ draining lymph nodes expressed the co-stimulatory ligand CD40 within 6 days post-infection. Results indicate that NADPH oxidase 1 modulates the innate and adaptive cellular immune response to influenza virus infection, while also playing a role in host survival. Results suggest that NADPH oxidase 1 inhibitors may be beneficial as adjunct therapeutics during acute influenza infection.

## Introduction

Despite extensive influenza virus surveillance and seasonal influenza vaccination coverage, influenza A virus (IAV) remains a major threat to public health. Seasonal influenza viruses cause illness in 2–5 million individuals annually, and 250,000–500,000 will succumb to complications from the disease [1]. Furthermore, the continual reassortment of IAVs within wild birds and domestic animals drives the occasional emergence of avian or swine influenza viruses that can infect humans [2, 3]. Some of these prove to be highly pathogenic, such as H5N1 and H7N9, which are fatal in 20–60% of individuals [4]. In the majority of lethal cases of influenza, death is attributed to acute respiratory distress syndrome [5], a more severe form of acute lung injury [6]. Current efforts to combat death related to IAV infection target the virus: vaccination and antiviral therapy. Both of these approaches are vulnerable to loss of efficacy due to viral mutations [7, 8]. Furthermore, several lines of investigation have implicated the host immune system as a contributing factor to pathology [9–11]. Along with vaccination and antivirals, there has been interest in development of adjunct therapeutics to decrease the inflammatory processes that underlie acute lung injury/acute respiratory distress syndrome by targeting the host immune system [12–14]. Such a strategy has been shown to improve outcomes of IAV infection in mouse models [10, 15, 16] and in the clinic [17]. These results underscore the potential of adjunct therapeutics to decrease the disease burden of IAV.

Reactive oxygen species (ROS) have been implicated in the lung pathology associated with severe cases of seasonal or pandemic IAV [18–24]. Superoxide produced by NADPH oxidase 2 (Nox2) has been shown to contribute to influenza-mediated lung pathology [23, 25, 26]. However, other sources of ROS in the lung include the Nox1 and Nox4 isoenzymes, as well as the closely related dual oxidase enzymes (Duox1 and Duox2), all of which are expressed by alveolar epithelial cells [27, 28]. In a previous study, Nox1 was shown to modulate influenza-induced inflammation in the early phase (days 3–7) post-infection (p.i.) with a non-lethal dose of influenza [29]. However, the influence of Nox1 after a lethal challenge of influenza infection has not been reported. It is also unclear how Nox1 might contribute to the development of adaptive immune responses following influenza virus clearance. In this study, we demonstrate that mice expressing an inactive form of Nox1 (Nox1*^/Y^ mice) [30] have improved survival after IAV challenge compared with C57BL/6 control mice. We also observed alterations to the adaptive immune response after IAV challenge, including a decreased percentage of virus-specific CD8^+^ T cells in the lungs, an increased percentage of virus-specific CD8^+^ T cells expressing the IL-7 receptor (CD127) in the lungs and draining lymph nodes, and an increased percentage of T cells in the lung and spleen with cytokine effector function *ex vivo* in Nox1*^/Y^ mice. These differences were associated with increased CD40 expression on the dendritic cells (DCs) of the lung-draining lymph node (dLN) in Nox1*^/Y^ mice. Our results suggest that Nox1 may negatively influence the optimal development of the early adaptive immune response to IAV infection.

## Results

### Nox1 contributes to PR8-induced morbidity and mortality

**Fig 1:**
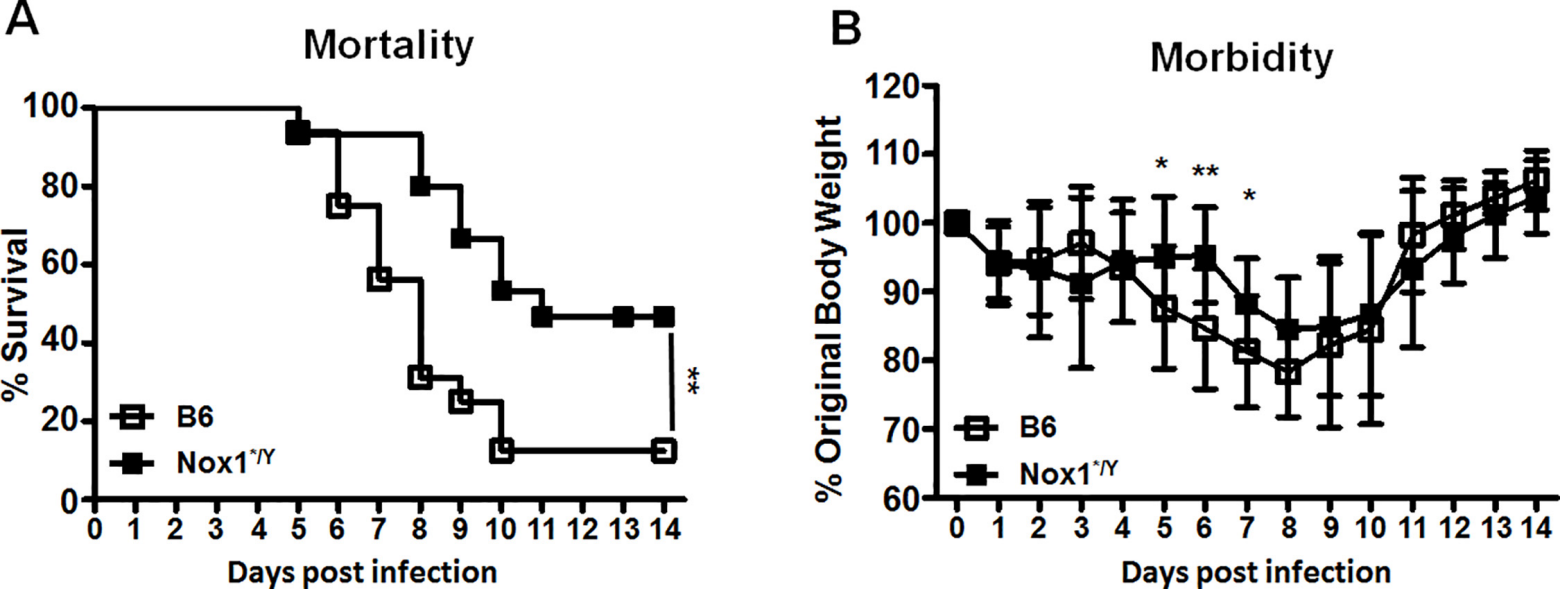
Nox1*^/Y^ mice have improved survival and delayed weight loss following intranasal challenge with IAV. B6 and Nox1*^/Y^ mice were challenged with 50 MID_50_ IAV. Mortality is plotted in A as percentage of mice surviving over time. A significant difference (*p*<0.01) was observed between the B6 and Nox1*^/Y^ mortality curves by both the Log-rank test and the Gehan-Breslow-Wilcoxon Tests. Morbidity is plotted in B as percentage of weight on day of infection (day 0). Results represent mean values ± standard deviation from the mean (S.D.). Average percent original body weights were compared between B6 and Nox1*^/Y^ mice at individual days p.i. by a Student’s *t* test. Data compiled from two independent experiments; *n* = 15–16 mice per group (*, *p* < 0.05; **, *p* = 0.01).

In a previous report, using mice lacking Nox1 gene, Selemidis et al. [29] reported that Nox1^-/Y^ mice had increased weight loss at day 3 p.i. along with increased inflammatory mediator gene expression. However, they also observed that by day 7 p.i. the Nox1^-/Y^ mice had decreased inflammatory mediators compared with B6 controls. Furthermore, virus-specific cellular immunity, weight loss and survival after day 7 have not been previously addressed. Since studies with mice lacking the entire Nox1 gene eliminates a number of possible protein-protein interactions, specifically with p22phox, NOXO1, NOXA1, and Rac1, which can proceed normally when only the active site is affected, we used mice lacking catalytically active domain of Nox1 (S1 Fig), and compared morbidities and mortalities of influenza A virus infected Nox1*^/Y^ mice with B6 mice. Nox1*^/Y^ mice and B6 control mice were infected with PR8 intranasally at 50 MID_50_. As shown in Fig 1, Nox1-deficiency provided a marked increase (3.7-fold) in survival following infection (Fig 1A). As expected, both B6 and Nox1*^/Y^ mice showed loss of body weight due to IAV infection, but Nox1*^/Y^ mice demonstrated a delay in weight loss between day 4 and 8 p.i. (Fig 1B). Similar results were observed when animals were challenged with PR8 virus at 1 LD_50_ (data not shown). These data suggest that Nox1 contributes to the morbidity and mortality of PR8 influenza virus infection.

### Nox1 deficiency leads to altered T cell phenotypes after PR8 infection

The differences observed in morbidity and mortality (Fig 1A and 1B) between Nox1*^/Y^ and B6 mice appeared no earlier than day 5 p.i. This coincides with the time at which PR8-specific CD8^+^ T cells migrate to the lungs from the lung draining lymph nodes (dLN) [31]. This prompted us to analyze the phenotype of the T cells arising during PR8 infection. Mice were infected with a sub-lethal dose of virus, 20 MID_50_, to allow them to survive long enough to develop adaptive immune responses. We isolated dLN from B6 and Nox1*^/Y^ mice at day 3, 6, 9 and 15 p.i., as well as lungs and spleens at day 9 and 15 p.i. The time points were chosen to permit observation of the development of the T cell response in the dLN and the peak of the T cell migration to the lung [31].

**Fig 2:**
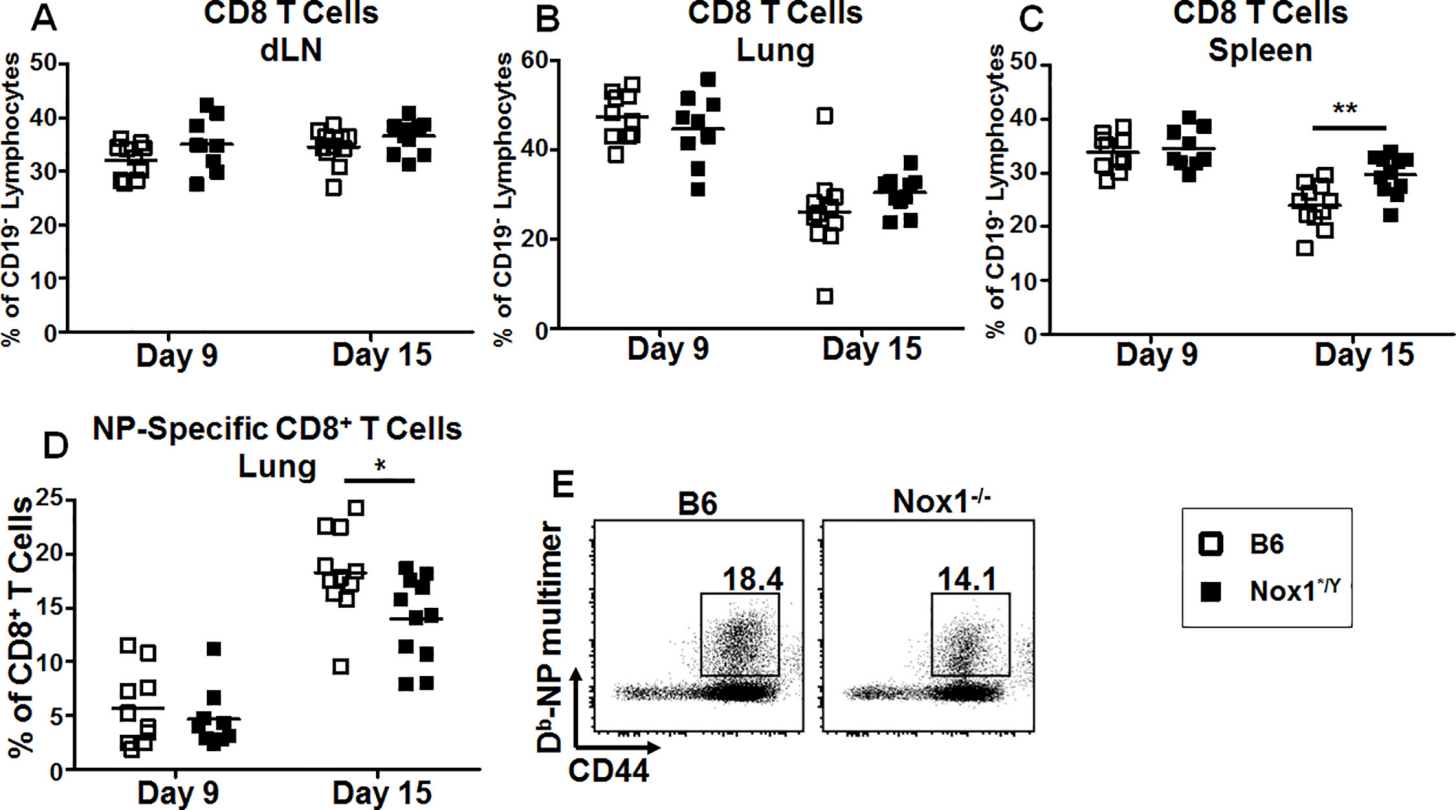
Lack of Nox1 catalytic activity alters frequencies of CD8^+^ T cells after IAV infection. Single-cell suspensions of dLN (A), lung (B, D, E) and spleen (C) harvested from B6 and Nox1*^/Y^ mice at day 9 and 15 p.i. were stained with antibodies and D^b^-NP-IAV pentamer then analyzed by flow cytometry. CD19^+^ cells were excluded from singlet lymphocyte events before differential CD4^+^ and CD8^+^ gating. NP multimer^+^ cells were gated from CD8^+^ cells as demonstrated in the representative FACS dot plots in E. E gated on the day 15 lung CD8^+^ T cell populations. Results in A-D plotted as individual mice with the horizontal bar indicating the mean. 2-way ANOVA indicates that the Nox1*^/Y^ genotype affects the percentage of CD8^+^ T cells plotted in A (*p* < 0.05), C (*p* < 0.01) and D (*p <* 0.05). Bonferonni post-test indicates significant difference between B6 and Nox1*^/Y^ mice at day 15 p.i. for C and D. Data compiled from two independent experiments; *n* = 9–11 mice per group (*, *p* < 0.05; **, *p* < 0.01).

We first analyzed the total T cell frequencies in the dLN, lungs and spleens. There was no difference in the frequency of CD4^+^ T cells between Nox1*^/Y^ and B6 mice in any tissue (data not shown). On average, the Nox1*^/Y^ genotype was associated with a higher percentage of CD8^+^ T cells in the dLN at day 9 and 15 after PR8 infection (Fig 2A), although no consistent difference was observed at day 3 or 6 p.i. (data not shown). There was no difference in the percentage of CD8^+^ T cells in the lungs (Fig 2B). Also, the percentage of CD8^+^ T cells was modestly but significantly increased in the spleens of Nox1*^/Y^ mice by day 15 p.i. (Fig 2C). However, there was no difference in CD4^+^ or CD8^+^ T cell frequency between naïve Nox1*^/Y^ and B6 mice (data not shown). We next investigated the frequencies of IAV-specific CD8^+^ T cells using a D^b^-IAV-NP pentamer. We observed a significant decrease in the percentage (Fig 2D and 2E), but not the absolute number (data not shown), of NP-specific CD8^+^ T cells in the lungs of Nox1*^/Y^ mice at day 15 p.i. No differences were observed in the NP-specific CD8^+^ T cell responses of the dLN or spleen (data not shown). To summarize, an increased percentage of CD8^+^ T cells was seen in the dLN of Nox1*^/Y^ after PR8 infection, with increased CD8^+^ T cells in the spleens of Nox1*^/Y^ mice by day 15 p.i. However, this correlated with a decreased frequency of NP-specific CD8^+^ T cells in the lungs at day 15 p.i.

**Fig 3:**
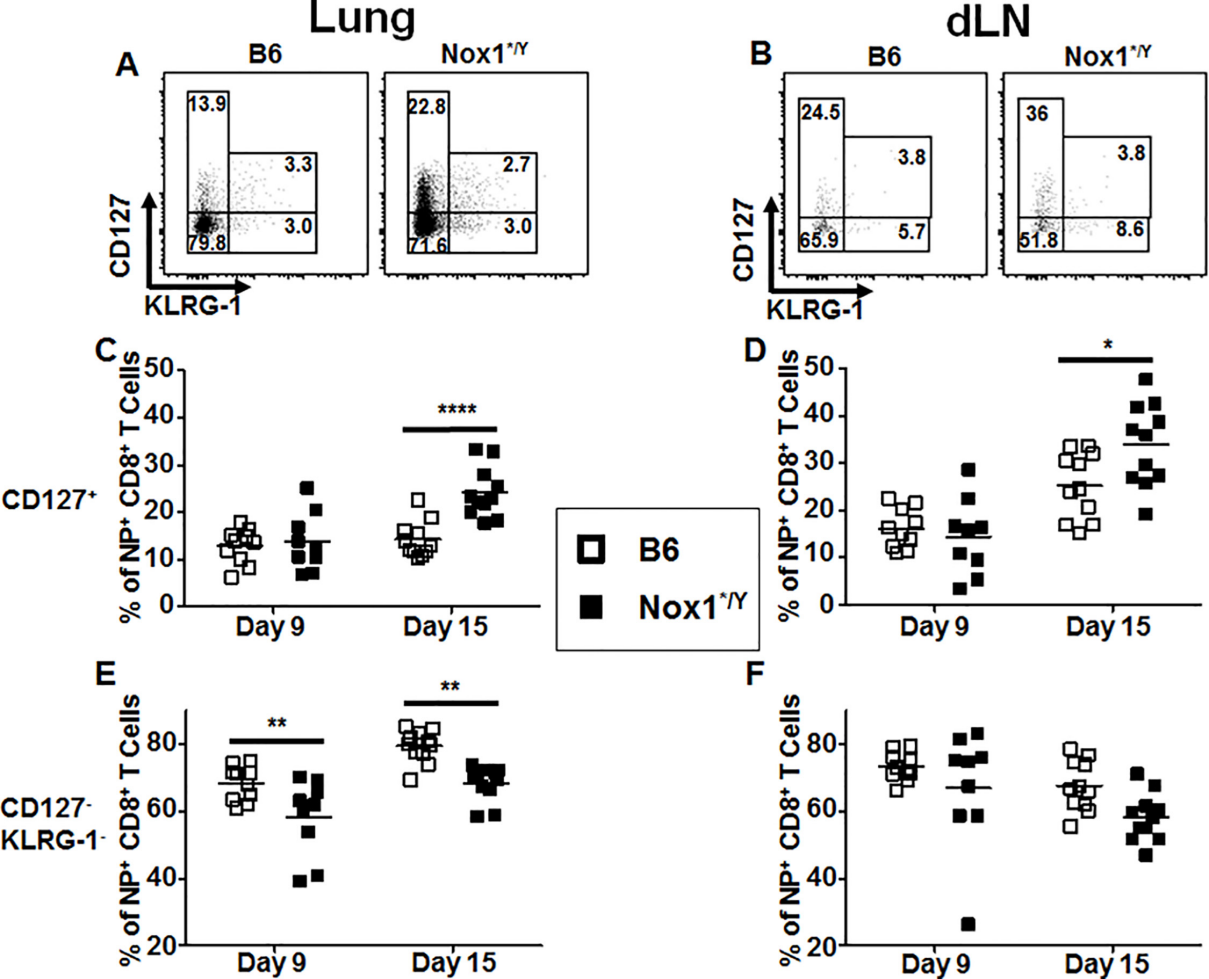
Nox1*^/Y^ mice have a greater percentage of CD127^+^ NP-specific CD8^+^ T cells at day 15. Single-cell suspensions of lung (A, C, E) and dLN (B, D, F) harvested from B6 and Nox1*^/Y^ mice at day 9 and day 15 p.i. were stained with antibodies and D^b^-NP-IAV pentamer then analyzed by flow cytometry. CD19^+^ cells were excluded from singlet lymphocyte events before differential CD4^+^ and CD8^+^ gating. CD127 and KLRG-1 staining was analyzed on D^b^-NP-IAV pentamer^+^ cells among CD8^+^ cells as demonstrated in the representative FACS dot plots taken from day 15 p.i. samples in A and B. Results in C-F plotted as individual mice with horizontal bar indicating mean. 2-way ANOVA indicates that the Nox1*^/Y^ genotype contributes to increased lung CD127^+^ cells (C, *p* < 0.001) and decreased double-negative CD127^-^KLRG-1^-^ expressing cells (E, *p* < 0.0001; F, *p* < 0.05). Bonferonni post-test indicates significant difference between B6 and Nox1*^/Y^ mice at day 15 p.i. for C and D, and at both day 9 and day 15 for E. Data compiled from two independent experiments; *n* = 9–11 mice per group (*, *p* < 0.05; **, *p* < 0.01; ****, *p* < 0.0001).

Having observed a modest difference in the percentage of NP-specific CD8^+^ T cells in the lungs between Nox1*^/Y^ and B6 mice, we next investigated the phenotypes of these cells based on surface protein expression. During the expansion of a CD8^+^ T cell response after antigen stimulation, the progeny cells acquire genetic programming which directs their fate during the contraction of the response. In the mouse models of infection with vesicular stomatitis virus or *Listeria monocytogenes*, surface expression of the IL-7 receptor (CD127) identifies cells fated to survive contraction and develop into the memory population [32, 33]. Also, surface expression of KLRG-1 identifies cells with a decreased propensity to replicate, and that are more likely to undergo programmed apoptosis during the contraction of the CD8^+^ T cell response (reviewed in [33]). No change was noted in the percentage of NP-specific CD8^+^ T cells expressing KLRG-1 in the lung (Fig 3A), dLN (Fig 3B), and spleen (data not shown). However, we observed a significant increase in cells expressing CD127 in both the lung (Fig 3A and 3C) and dLN (Fig 3B and 3D) of Nox1*^/Y^ mice at day 15 p.i. Cells expressing neither CD127 nor KLRG-1 were correspondingly decreased in these tissues (Fig 3A, 3B, 3E and 3F) from Nox1*^/Y^ mice. In summary, the NP-specific CD8^+^ T cells in Nox1*^/Y^ mice express more CD127 than those in B6 mice at day 15 p.i.

**Fig 4:**
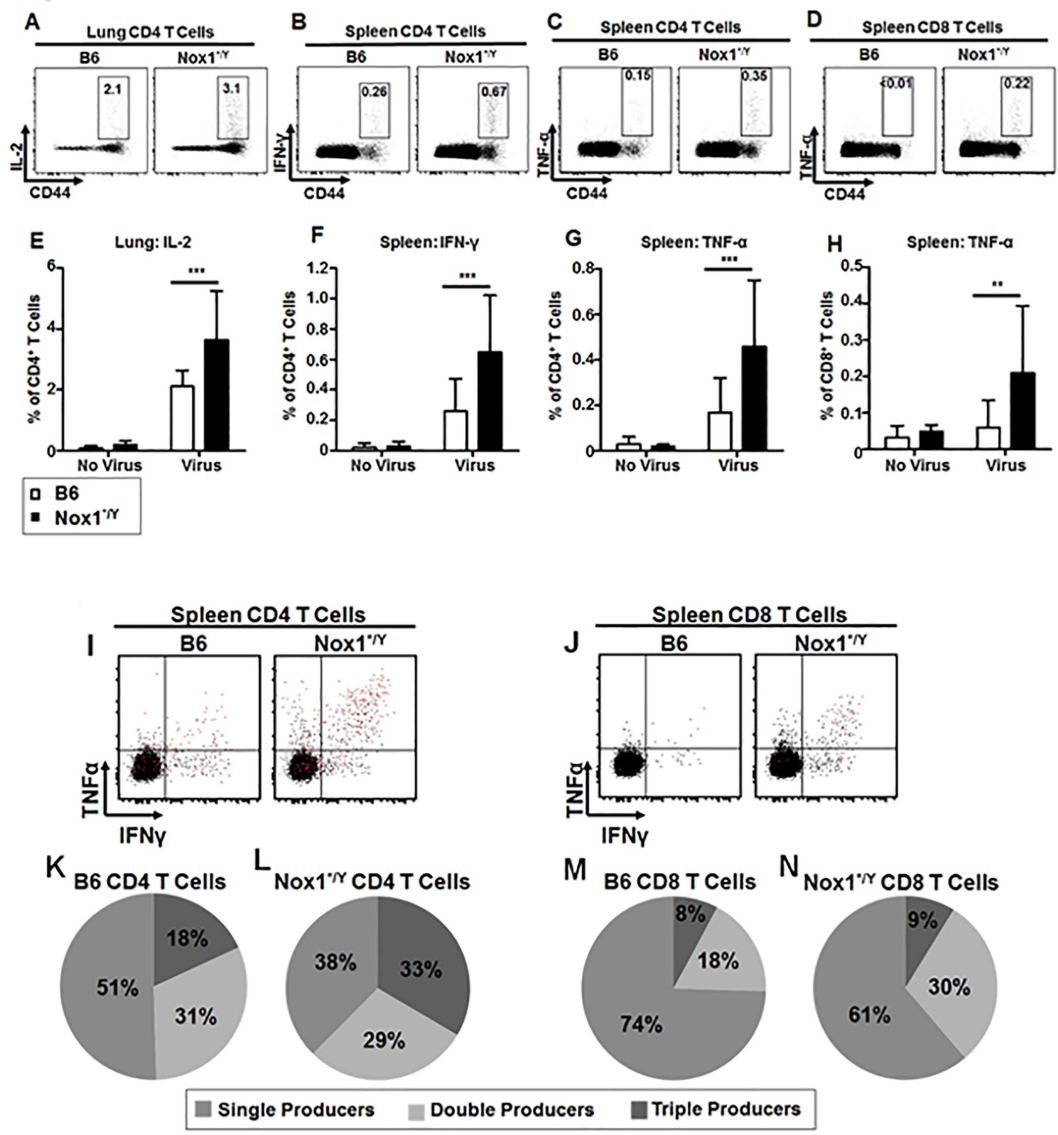
Nox1*^/Y^ mice have increased IAV-specific cytokine-producing T cells at day 15. Single-cell suspensions of lung (A, E) and spleen (B-D, F-N) harvested from B6 and Nox1*^/Y^ mice at day 15 p.i. were incubated overnight in the presence or absence of IAV at 1 MOI. Next, cells were stained extracellularly and intracellularly with antibodies and analyzed by flow cytometry. CD4^+^ and CD8^+^ populations gated from singlet lymphocyte events. A-D, representative dot plots of virus-stimulated B6 or Nox1*^/Y^ cells gated on the indicated T cell population. E-H, individual cytokines presented as a percent of the CD4^+^ or CD8^+^ populations. Results represent mean values ± S.D. I-J, representative dot plots of virus-stimulated B6 or Nox1*^/Y^ cells gated on the indicated T cell population. Red dots are IL-2^+^. K-N, data from Boolean gating of cytokines gated as in A-D presented as a percent of total cytokine (IFN-γ, TNF-α or IL-2)-producing CD4^+^ or CD8^+^ T cells. 2-way ANOVA indicates that the Nox1*^/Y^ genotype contributes to increased cytokine-producing cells after *ex vivo* virus stimulation in E-H (*p* < 0.01). Bonferroni post-test indicates significant difference between stimulated cells from B6 and Nox1*^/Y^ mice for E-H. Data compiled from two independent experiments; *n* = 11 mice per group (**, *p* < 0.01; ***, *p* < 0.001).

Antigen-specific T cells produce cytokines upon activation by cognate antigen encounter [34–36]. To determine whether Nox1 affected the cytokine production capacity of T cells responding to IAV challenge, we compared the cytokine production of PR8-specific T cells from Nox1*^/Y^ mice with those from B6 mice by *ex vivo* intracellular cytokine assay. While no differences were seen at day 9 p.i. (data not shown), an increase was seen in the percentage of Nox1*^/Y^ lung CD4^+^ T cells which produced IL-2 after overnight stimulation with PR8 (Fig 4A and 4E). We also observed increases in both the percentages of splenic CD4^+^ T cells from Nox1*^/Y^ mice able to produce IFN-γ and those able to produce TNF-α (Fig 4B, 4C, 4F and 4G). Furthermore, a higher percentage of CD8^+^ T cells in Nox1*^/Y^ spleens produced TNF-α than B6 controls (Fig 4D and 4H). We further analyzed the cytokine effector function of the CD4^+^ and CD8^+^ T cell populations by Boolean gating our multiparameter flow cytometry data as described by Seder *et al*. [37]. We observed that a greater percentage of splenic T cells from IAV-infected Nox1*^/Y^ mice were able to produce more than one cytokine in response to antigen at day 15 p.i. Specifically, Nox1*^/Y^ mice had a greater percentage of triple cytokine-producing CD4^+^ T cells in their spleens than B6 controls (Fig 4I, 4K and 4L). Nox1*^/Y^ mice also had a greater percentage of double cytokine-producing CD8^+^ T cells in their spleens (Fig 4J, 4M and 4N). Together, these results indicate that by day 15 p.i., Nox1*^/Y^ mice have both a greater percentage of PR8-specific T cells which have cytokine effector function, and a greater percentage of PR8-specific T cells which are cytokine multipotent, in response to antigen stimulation.

### No effect on B cell responses observed with Nox1 deficiency

**Fig 5:**
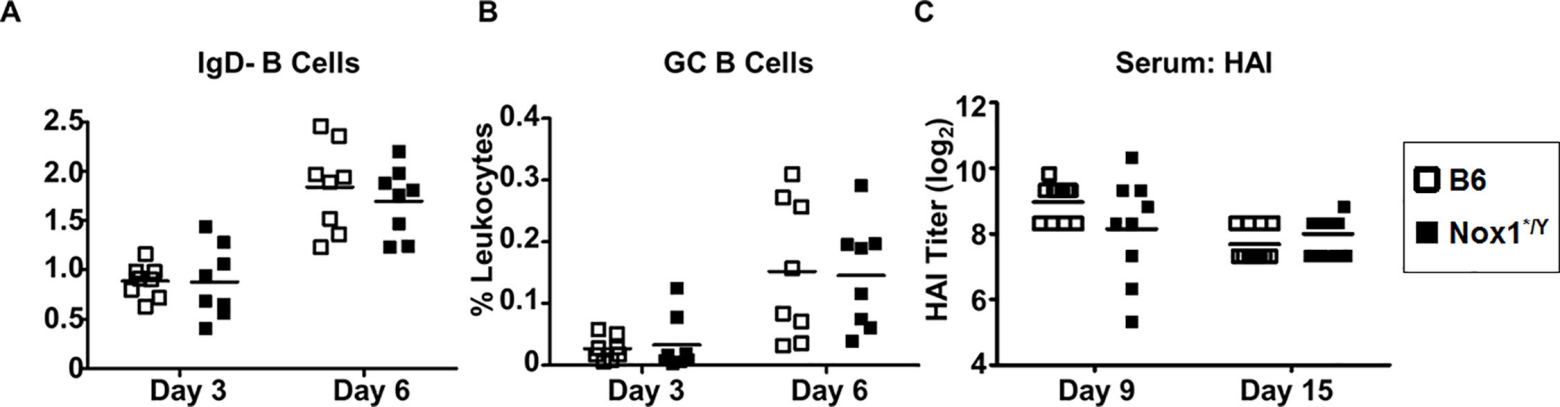
Germinal center B cell frequencies and HAI titers unaffected by lack of Nox1 catalytic activity. Single-cell suspensions of dLN (A-B) harvested from B6 and Nox1*^/Y^ mice at day 3 and 6 p.i. were stained with antibodies and analyzed by flow cytometry. A: B220^+^ IgD^-^ cells were gated from singlet leukocyte events. B: GC B cells were identified as the CD38^-^ GL7^+^ population among IgD^-^ B cells. C: day 9 and 15 serum titers of HA-binding antibody as determined by HAI assay. Results in A-C plotted as individual mice with horizontal bar indicating mean. Data compiled from two independent experiments; in A & B, *n* = 8 mice per group; in *D*, *n* = 9–11 mice per group.

The antibody-mediated adaptive immune response to IAV is the most important correlate of protection from disease [38]. CD4^+^ T cells provide help to B cells to produce the optimal quantity and quality of antibodies after antigen exposure [39, 40]. Having observed increased cytokine production from CD4+ T cells after *ex vivo* stimulation in the spleen and lung of Nox1*^/Y^ mice, we investigated the B cell response for corresponding changes. We observed no consistent difference in the frequency or number of PR8-specific IgM or IgG producing B cells as measured by ELISpot at day 9 or 15 p.i. (data not shown). B cells are thought to encounter IAV antigen in the dLN as early as day 3 p.i., and class-switched B cells are apparent within the first week p.i. [41]. Therefore, we queried the surface phenotypes of the B cells in the dLN at day 3 and 6 p.i. As determined by FACS analysis, the frequency of IgD^-^ class-switched B cells (Fig 5A) and germinal center B cells (Fig 5B) was unchanged in the dLN at day 3 to 6 after IAV infection. We did not observe any difference in hemagglutination inhibition (HAI) titers at day 9 or 15 (Fig 5C). Overall, we observed little impact of Nox1 deficiency on the magnitude of the B cell-mediated early immune response to IAV.

### CD40 expression on DCs is enhanced at early time points in dLN of Nox1-deficient mice

**Fig 6:**
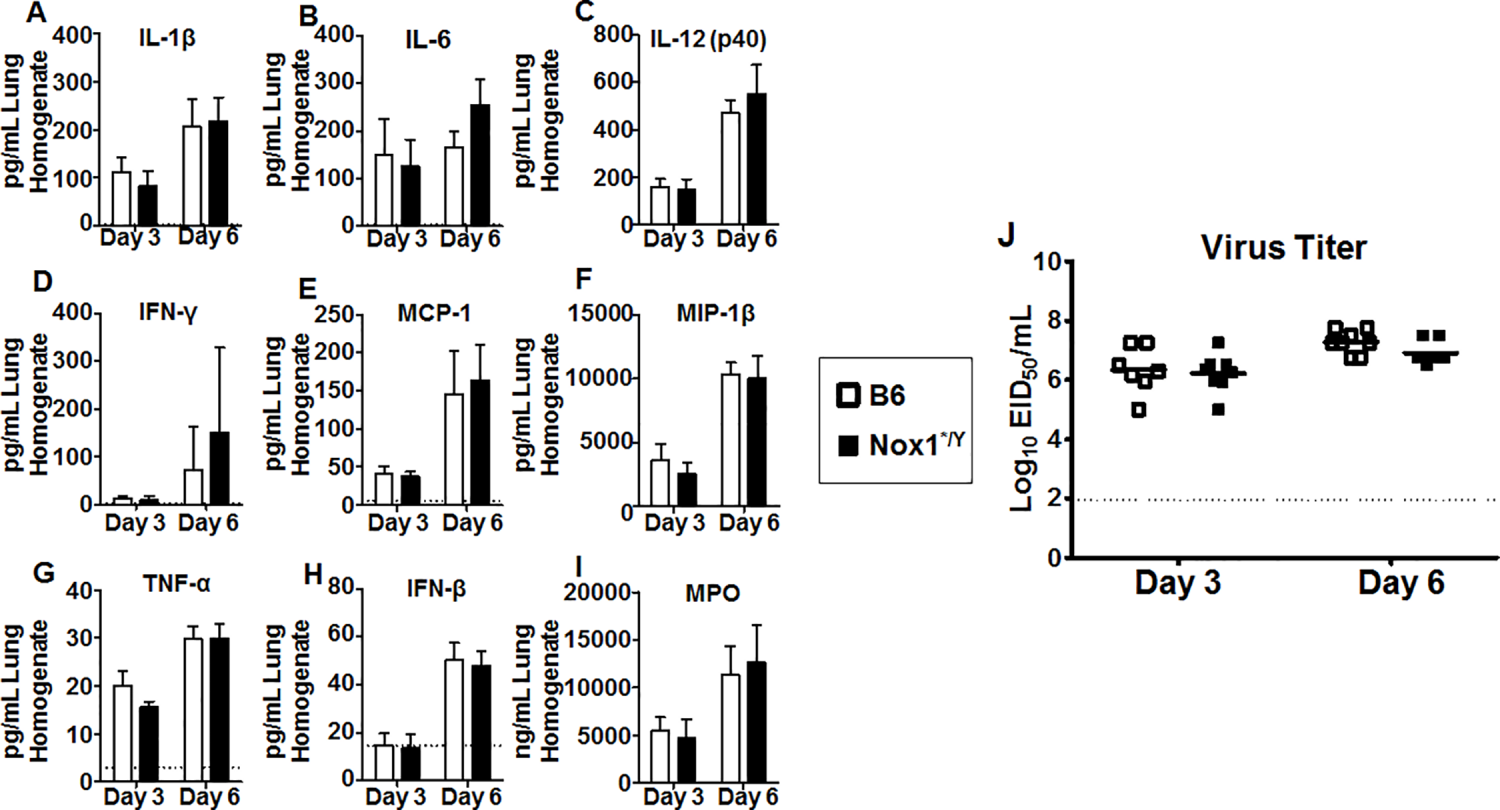
Lack of Nox1 catalytic activity does not affect lung inflammatory protein levels or viral titers. Supernatant harvested from whole lung homogenate was tested for cytokine and chemokine protein levels by Bioplex assay (A-G) and ELISA (H). Supernatant MPO protein levels were determined by ELISA (I). IAV titers were determined by EID_50_ assay (J). Assay limits of detection depicted by dashed horizontal line. Results in A-I represent mean values ± S.D. All samples run in duplicate; average of two wells used to generate final data. Result in J plotted as individual mice with horizontal bar indicating mean. Data compiled from two independent experiments, *n* = 7–8 mice per group.

**Fig 7:**
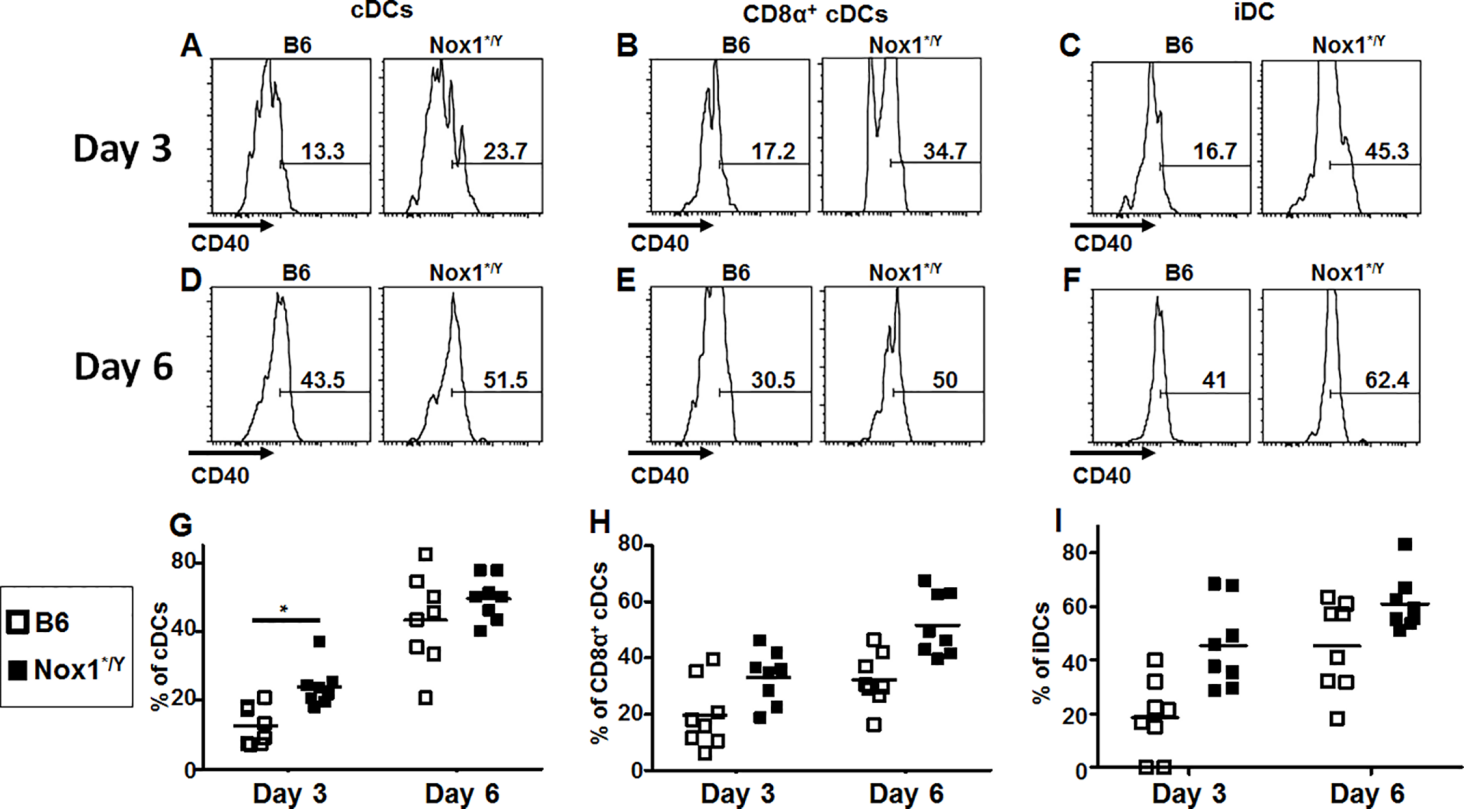
A greater percentage of cDCs and iDCs in the dLN of Nox1*^/Y^ mice express CD40. Single-cell suspensions of dLN harvested from B6 and Nox1*^/Y^ mice at day 3 and 6 p.i. were stained with antibodies and analyzed by flow cytometry. Among SSC^lo^ singlet lymphocytes, CD11c^hi^ B220^hi^ cells were gated for the pDC population. All other DC populations were gated from the B220^-^ cells using the gating strategy described by Ballesteros-Tato et al. [43], including tDCs (CD11c^+^ MHCII^hi^) from which were gated both the CD103^+^ and CD103^-^ tDC populations, cDCs (CD11c^hi^ MHCII^int^) from which were gated the CD8α^+^, CD4^+^ and double negative cDC populations, and iDCs (CD11c^int^ MHCII^int^). CD40 expression was analyzed on individual DC populations as shown in representative histogram plots (A-F). Results in G-I plotted as individual mice with horizontal bar indicating mean. 2-way ANOVA indicates the Nox1*^/Y^ genotype affects the percentage of populations graphed in G (p < 0.01), H and I (*p* < 0.001). Bonferonni post-test indicates significant difference between B6 and Nox1*^/Y^ mice at day 3 p.i. for G. Data compiled from two independent experiments; *n* = 9–11 mice per group (*, *p* < 0.05).

The differences we observed in the phenotype and function of T cells between Nox1*^/Y^ and B6 mice at day 15 after PR8 IAV infection may be linked to differences in the priming environment of the T cells [32, 33, 42]. Therefore, we investigated the early priming environment of the T cells in the lung and dLN. The lungs and dLN of mice were isolated at day 3 and 6 p.i. after the same sublethal dose of virus (20 MID_50_) at which the differences in T cell phenotypes were observed at day 15 p.i. Lungs were homogenized and queried for inflammatory markers and viral titers. As shown in Fig 6, no consistent difference was observed in markers of lung inflammation between B6 and Nox1*^/Y^ mice, including cytokines (IL-1β, IL-6, IL-12(p40), IFN-γ, TNF-α, and IFN-β), chemokines (MCP-1, MIP-1β), and myeloperoxidase protein (MPO). There was also no difference in lung viral titers (Fig 6J), nor apparent differences in the microscopic appearance of inflammation or in dissemination of virus in the lung tissue harvested at day 3 and 6 p.i between B6 and Nox1*^/Y^ mice (S2A and S2B Fig). In the dLN, there were no consistent differences in T or B cell populations at day 3 or 6 p.i (data not shown). No consistent differences were observed in the percentage of cells comprising the various dLN DC subsets, including neither the dLN resident DCs: interstitial DCs (iDCs), plasmacytoid DCs (pDCs), and conventional DCs (cDCs), nor the DCs which traffic from the lung tissue (tDCs) [43] (data not shown). We also did not observe a change in the frequency of CD86 expression among any of these populations (data not shown). However, an increased percentage of cells expressing CD40 was observed among certain DC subsets in the dLN in Nox1*^/Y^ mice. There was an increased frequency of total cDCs expressing CD40 at day 3 p.i. (Fig 7A and 7G), along with an overall increase in CD40^+^ cells among the CD8α^+^ cDC subset (Fig 7B, 7E and 7H) as well as the iDCs (Fig 7C, 7F and 7I) at the early time points. CD40 is expressed on activated DCs [44] and serves as a costimulatory ligand for activation of DCs by T cells [45–47]. These data suggest that although lack of Nox1 activity does not appear to affect the gross inflammation level in the lungs of PR8-infected mice after sublethal infection, it may alter costimulatory signals involved in T cell priming.

## Discussion

Reactive oxygen has long been proposed to play a role in inflammation including that of the lung. Several studies showed that Nox2 contributes to immunopathology following IAV infection [23, 25, 26]. The role of other Nox-family enzymes expressed in the lung [27] have been less thoroughly investigated. In our studies, Nox1*^/Y^ mice had a significant improvement in survival and morbidity assessed by weight loss after IAV challenge. At a sublethal dose of virus, Nox1*^/Y^ mice also had altered T cell phenotypes by day 15 p.i. However, it is not clear that the phenotypic changes to the T cell population observed at day 15 p.i after a sublethal challenge are related to differences in morbidity and mortality observed as early as day 5 p.i. after a lethal challenge. At early time points, we failed to observe differences between wild-type and Nox1*^/Y^ mice by measurements of inflammation, nor did we observe a difference in lung viral titers. This suggests the effects of Nox1-generated ROS on survival may be more subtle, perhaps involving signaling pathways in the lung or in other organs. Consistent with this interpretation, at days 3 and 6 p.i. certain DC subsets within the draining lymph nodes had increased CD40 expression. Together, these results suggest that Nox1 plays a role in increasing mortality after IAV infection, perhaps related to or in parallel with the role of Nox1 in signaling events during priming of the early adaptive immune response.

To our knowledge, there is only one other published study investigating the role of Nox1 in IAV infection: using mice lacking the entire Nox1 gene, Selemidis, et al. [29] reported increased weight loss and increased inflammation in Nox1^-/Y^ mice as determined by histology, indicators of oxidative stress, and gene expression of inflammatory mediators. Furthermore, alterations to neutrophils and macrophages, but no changes to adaptive immune cell populations, recovered by bronchoalveolar lavage were reported. Importantly, there were several differences between our model system and that used by Selemidis, et al. The Selemidis, et al. study used Nox1^-/y^ mice which lack any Nox1 protein expression [48]. In contrast the Nox1-deficient mice used herein express a catalytically deficient Nox1 protein that is expressed under the control of its normal promoter [30]. This may result in some differences in phenotype. For example, the truncated protein may be capable of interacting with some of its normal protein binding partners such as p22*phox*, which shares interactions with other Nox isoforms (Nox2, Nox3, Nox4) [49, 50]. Also, Selemidis, et al. used HkX-31 IAV infection, an H3N2 laboratory strain of IAV, while we used PR8, an H1N1 laboratory strain of IAV that is known to be more virulent than HkX-31 in mice [51]. Finally, Selemidis, et al. investigated the cellular immune response to IAV in the BALF through day 7 p.i. Most of the differences we report herein are in spleen, draining lymph node, or whole lung, with important differences in T cell phenotypes not appearing until 15 days p.i. The different experimental approaches used might also be expected to generate differing results, including querying changes in the cytokine and chemokine responses by mRNA levels rather than protein levels, and sampling immune cell subsets from the BALF in contrast to whole, unperfused lung. Taken together, the differences in model systems and experimental approaches make comparisons difficult and may account for apparent contrasts between the two studies.

The mechanism for the increased survival of Nox1*^/Y^ mice compared with B6 controls in our study remains unknown. Excessive lung inflammation can exacerbate pathology [11] but convincing differences in measures of lung inflammation (inflammatory proteins, lung wet weights (data not shown) and histology), were not observed, consistent with the lack of difference in morbidity and mortality between the Nox1*^/Y^ and B6 mice before day 5 p.i. B-cell mediated IAV-specific antibody responses are the most important correlate of protection from IAV [38], and we were unable to observe any difference in B cell responses or antibody titers, which may explain the modest difference in morbidity and mortality observed. Virus is typically cleared from the mouse lung by day 6–8 p.i. [52–54], and we observed no difference in viral titers during this timeframe. Furthermore, when compared with B6 controls, Nox1*^/Y^ mice had equivalent percentages of NP-specific CD8^+^ T cells in their lungs at day 3, 6 (data not shown) and 9 p.i. This suggests that the alterations to T cell phenotypes arising by day 15 p.i. are likely to be consequences of earlier differences in T cell programming during the expansion phase, rather than contributing to improved survival of Nox1*^/Y^ mice through antiviral activity. Therefore, it is possible that other signaling pathways and resulting effector molecules induced by influenza virus infection of Nox1*^/Y^ mice may be responsible for increased survival. Further studies addressing the role of Nox1 in different cell types, both in the lymphoid organs and peripheral tissues, may help to explain the improved survival of Nox1*^/Y^ mice.

The surface phenotype of CD8^+^ T cells can be affected by the priming environment as well as the differentiation stage of the clone [32, 42]. For example, in the vesicular stomatitis virus and *Listeria monocytogenes* models, the ratio of CD127^+^ memory precursor effector cells to KLRG-1^+^ short lived effector cells is linked to the level of inflammation during priming, with lower inflammation favoring a larger proportion of memory precursor effector cells and higher inflammation skewing the response towards short lived effector cells [55]. In this study, a greater percentage of the NP-specific CD8^+^ T cells in the lung and dLN of Nox1*^/Y^ mice expressed CD127, which corresponded with a decrease in the frequency of CD127^-^KLRG-1^-^ T cells without a change in the KLRG-1-expressing cells. In the VSV and *L*. *m*. models, the double negative CD127^-^KLRG-1^-^ population is a precursor to both the CD127- and KLRG-1-expressing populations [32]. Therefore, increased CD127 expression on the D^b^-IAV-NP-specific CD8^+^ T cells may indicate that the NP-specific CD8^+^ T cells in Nox1*^/Y^ mice have advanced further in the process of differentiation by day 15 p.i. than their B6 counterparts. When the fact that there are fewer NP-specific CD8^+^ T cells in the lungs of Nox1*^/Y^ mice at this same time point is taken into account, we can speculate that this population is undergoing contraction earlier than the corresponding B6 population. However, the contraction of the CD8^+^ T cell response is mediated by cytokine signals [56]. No differences were seen between B6 and Nox1*^/Y^ mice in lung cytokine expression at day 3 or 6 p.i., but the levels of cytokines in the lungs or dLN at later time points p.i. were not measured.

Production of cytokines is one of the effector functions of an activated T cell [34, 57]. Production of IFNγ, TNFα and IL-2 have been shown to be hierarchical in the IAV-specific response, with IFN-γ being produced by most IAV-specific CD8^+^ T cells, of which a subset can produce TNF-α, and only the most functionally differentiated cells produce IL-2 [37, 58]. CD4^+^ T cells can also produce all three cytokines after IAV infection [36], and among influenza-specific CD4^+^ T cells in human blood, triple cytokine producers are functionally superior to single-producers [59]. A larger percentage of CD4^+^ T cells in the Nox1*^/Y^ spleens produced all three cytokines, while a larger percentage of CD8^+^ T cells in Nox1*^/Y^ spleens produced two of these cytokines. CD4^+^ T cells, with their known T-helper function, are expected to produce higher levels of cytokines than the CD8^+^ T cells [60]. When viewed alongside the increased CD127 expression of NP-specific CD8^+^ T cells in the lung and dLN, the increased percentage of cytokine-producing IAV-specific T cells in the lung and spleens suggests that by day 15 p.i. Nox1*^/Y^ mice have a T cell response that would be better able to respond to IAV re-challenge.

Changes in the phenotype and cytokine-producing functions of T cells in IAV-infected mice compared to B6 controls raises the question of the relationship between Nox1-expressing cell types and mechanisms that influence T cell priming. Earlier studies in murine model [29] and in human lung [61] have shown Nox1 expression in alveolar epithelial cells, bronchial cells, and endothelium. Results from our preliminary studies showed an increase in Nox1mRNA expression in IAV-infected human epithelial (A549), endothelial (HULEC) and monocytic (THP-1) cell lines (data not shown). Furthermore, when compared with lung tissue from naïve mice, IAV (A/PR8 and A/Mexico/4108/2009)-infected Nox1*^/Y^ mice show increase in Nox1mRNA expression at 24h and 48h p.i. (data not shown). Therefore it is possible that virus replication in the respiratory epithelium and the ensuing cytokine/chemokine milieu may induce an increase in Nox1 expression in lung resident cells and lung infiltrating antigen presenting cells that may alter virus dissemination, antigen presentation and T cell priming. Future studies are planned to address this possibility.

In the context of influenza infection, the co-stimulatory molecule CD40 has a role in helping both the CD4^+^ and CD8^+^ T cell responses. CD40 has been shown to help the influenza-specific CD8^+^ T cells survive premature contraction mediated by Tregs [62]. It is interesting to note that although we see increased CD40 on Nox1*^/Y^ dLN DCs, and increased CD127 expression and decreased CD127^-^KLRG-1^-^ NP-specific CD8^+^ T cells in the lungs and dLN of Nox1*^/Y^ mice at day 15 p.i., we also saw a decreased frequency of NP-specific CD8^+^ T cells in the lungs. This emphasizes the importance of determining the role of Nox1 in the contraction of the IAV-specific CD8^+^ T cell response. CD40 is also involved in the self-help feedback loop of influenza-specific CD4^+^ T cells, in which previously primed CD4^+^ T cells license DCs to better prime the next generation of CD4^+^ T cells [63]. The increased percentage of IFNγ and TNFα produced by CD4^+^ T cells from the spleen after *ex vivo* virus re-stimulation indicate a greater percentage of IAV-specific CD4^+^ T cells are present [63]. This suggests that some aspect of this CD4^+^ T cell feedback loop operates more efficiently in Nox1*^/Y^ mice than in B6 mice.

ROS are generated in a controlled manner via the activity of Nox enzymes in most or all tissues, and play roles in both normal physiology and disease [50, 64]. We demonstrate here that normal expression of native, catalytically intact Nox1 enzyme results in increased mortality after IAV infection, accompanied by earlier and more severe weight loss compared to mice expressing catalytically-inactive Nox1. Nox1*^/Y^ mice appeared to have improved T cell responses over normal mice including increased CD127 expression and *ex vivo* cytokine production by day 15 p.i. This was associated with increased CD40 expression on DC subsets in the dLN at day 3 and 6 after infection. Our results suggest that Nox1 activity contributes to mortality at the peak of influenza virus infection, while having an inhibitory effect on development of the early adaptive immune response. Although some ROS inhibitors have shown immunomodulatory effects [19], based on our findings, the use of Nox1-selective inhibitors should be explored as an adjunct therapy (e.g., along with antivirals) to decrease the mortality and morbidity associated with influenza.

## Materials and Methods

### Mice and virus infections

Mice expressing catalytically inactive Nox1 were generated by crossing Nox1^*loxp/loxp*^ mice (described in [30]) with mice expressing the germline specific Zp3-cre. Because Nox1 is encoded on the X chromosome, the offspring of these crosses were bred to generate Cre-expressing, and therefore Nox-truncated, hemizygous males (designated as Nox1^*/y^). Second mating was required between the males derived from the Cre/floxed Nox1 and females of same cross to generate females that were homozygous Nox1-inactive mice, which were continuously mated with the Nox1-inactive male mice. Schematic representation of exon 13, lac Z insertion site, primer sets used for genotyping of mice and beta-galactosidase expression is shown in S1A–S1C Fig. The colony of Nox1-modified mice was maintained at Charles River (Wilmington, MA). Four to six week old male Nox1*^/Y^ and control C57Bl/6 (B6) mice were housed in a specific pathogen-free environment in an Association for Assessment and Accreditation of Laboratory Animal Care International-accredited facility at the Centers for Disease Control and Prevention under the guidance of the Centers for Disease Control and Prevention’s Institutional Animal Care and Use Committee (IACUC, animal welfare assurance number A4365-01). The studies performed were approved by the IACUC (approved Protocol Number: 2406).

A/Puerto Rico/8/34 (PR8) virus was grown and stored as described [65]. PR8 was titrated for 50% lethal and 50% mouse infectious doses (LD_50_ and MID_50_, respectively) by administering serial ten-fold dilutions of egg-grown virus stock to 6-week old female B6 mice. Lethality, as defined by loss of greater than 25% original body weight, or infection, defined by positive Egg Infectious Dose (EID) titers in the lungs at day 3 p.i., were used as endpoints to determine LD_50_ or MID_50_ titers respectively, in the method described by Reed and Muench [66]. Age-matched mice were infected intranasally at 5–8 weeks of age with 50 MID_50_ or 20 MID_50_ of PR8 under anesthesia (Avertin; Sigma-Aldrich). Since the virus stock dilutions required for preparing a virus dose/inoculum (50μl) of 50 MID_50_ and LD_50_ varied by less than 2-fold dilution, clinical symptoms and lethality was evident at the calculated dose of 50 MID_50_. As per approved IACUC protocol, animal suffering and distress was minimized by following AALAS approved trainings for handling and care of animals. IACUC approved anesthetic was used during virus infection of mice. However, analgesics and anesthetics were not used during the observation period since both analgesics and anesthetics can interfere with outcomes such as inflammation and immune response to influenza infection [67]. The animals were monitored twice daily and there were no unexpected deaths during the observation period. In the survival studies, mice which lost more than 25% original body weight were humanely euthanized using IACUC protocol approved method, i.e., cervical dislocation under Avertin anesthesia. Mice which never dropped below 100% of original body weight were presumed to be uninfected and were omitted from longitudinal studies.

### Genotyping of Nox1*^/Y^ mice

Tail genomic DNA was prepared using Wizard® Genomic DNA Purification Kit (A1120, Promega). PCR was performed using following primer sets; FP-GTACTGCTCTACTCTTACAGG; RP1- GCAAGTGTCAGCCAGCAA; RP2-CTTCGCTATTACGCCAGCTG. Amplification was done using Redextract-N-Amp (R4775, Sigma) with the following PCR program; 95°C, 3 min, 1 cycle; 95°C, 30 sec; 52°C 20 sec; 72°C, 20 sec for 35 cycles; 72°C, 3 min followed by hold at 4°C. PCR products were analyzed by running 5μl on an ethidium bromide agarose gel (1.5%) and visualized by UV light. As a marker of gene expression, tongue harvested from wild type and Nox1*^/Y^ mice were screened for beta-galactosidase activity using standard protocol. Briefly, specimen was fixed in 10mM periodate-lysine-4% paraformaldehyde followed by 3x wash with PBS and incubation in a solution containing 20 mM dithiothreitol, 150 mM Tris Base, and 20% ethanol for 45 min at RT. After 3x wash with PBS, specimen was incubated in solution containing 2 mM X-gal, 4 mM potassium ferricyanide, 4mM potassium ferrocyanide, 2 mM MgCl_2_ in PBS, overnight at 4°C.

### FACS Analysis

Tissues were collected and processed to single-cell suspensions as follows: lungs were homogenized by incubation with collagenase I (263 U/ml) and lymphocytes were isolated by density gradient centrifugation (Lymphoprep; Stemcell Technologies); dLN and spleens were gently dissociated and incubated in collagenase IV (297 U/ml) for 30 min before filtering through a 40μM cell strainer followed by incubation in Red Blood Cell Lysis Buffer (Sigma-Aldrich) for 3–5 min. For surface stains, 1x10^6^ cells were first blocked with anti-mouse FcBlock (BD Pharmingen) and then stained with the following antibodies: anti-KLRG-1, anti-CD44, anti-CD103, anti-CD11c, anti-CD80, anti-CD138, anti-CD3, (Biolegend), anti-CD4, anti-CD127, anti-CD8α, anti-CD86, anti-B220, anti-MHC Class II, anti-CD11b, anti-CD69, anti-CD38, anti-IgD (eBioscience), anti-CD19, anti-CD40, anti-Ly6C, anti-GL7, and anti-CD86 (BD Pharmingen), as well as with R-PE labelled H-2D^b^ ASNENMETM (D^b^-NP-IAV) pentamer to label IAV nucleoprotein (NP)-specific T cell receptors (ProImmune). For intracellular cytokine staining, cells were incubated at 37°C with or without 1 multiplicity of infection (MOI) PR8 for 5 hours before overnight addition of GolgiStop and GolgiPlug at 1:1000 (BD Biosciences). Cells were washed twice with PBS before staining with anti-CD44, anti-CD8α and anti-CD4, permeabilized with BD Cytofix/Cytoperm, then stained with anti-IFN-γ, anti-TNF-α (Biolegend), and anti-IL-2 (ebioscience). Events were collected on a BD LSRII flow cytometer.

### ELISpot Assay

MAIPS4510 ELISpot plates (Millipore) were coated with either 100 HA units of PR8, or 0.5 μg of goat anti-mouse unlabeled human adsorbed anti-IgM or -IgG (Southern Biotech) overnight at 4°C. Plates were blocked with media supplemented with FBS and washed with 1X PBS-TWEEN20 (HyClone). 1.5–3 x 10^6^ splenocytes (prepared as for FACS analysis, above) were added to the plate followed by serial 3-fold dilutions. Plates were incubated overnight at 37°C. Biotinylated anti-mouse IgG or IgM (Southern Biotech) was added at 1:1000 dilution followed by alkaline phosphatase-streptavidin (Southern Biotech) at 1:1000 dilution. ELISpot plates were developed with Vector Blue Substrate Kit (Vector Laboratories). Spots were counted using an ImmunoSpot Analyzer (CTL).

### Virus titers

Lungs were harvested and frozen on dry ice before storage at -80°C. Lung viral titers were determined by EID assay as previously described [65] and calculated using the Reed-Muench method as described in detail by Balish, et al. [68].

### Bioplex Assay and ELISA

Lung supernatants (see Methods for ‘Virus titers’, above) were added in duplicate to a Bioplex plate (Bio-Rad) previously coated with anti-mouse antibody-conjugated beads specific for IL-1β, IL-6, IL-12 (p40), IFN-γ, MCP-1, MIP-1β, and TNF-α. Bioplex plate was processed and read according to manufacturer’s protocol on a Bioplex 200 Array Reader. Clarified supernatants were also added in duplicate to a pre-coated anti-mouse IFN-β ELISA plate (PBL Assay Science) and at a final dilution of 200-fold to an anti-mouse MPO ELISA plate (Hycult Biotech). ELISA plates were processed according to manufacturer’s directions and read at an absorbance of 450 nm on a BioTek Synergy 4 plate reader.

### Histology & Immunohistochemistry

Whole lungs were excised from mice and placed into 10% neutral buffered formalin. After 48 h, formalin was decanted and replaced with 70% ethanol. Fixed tissues were embedded in paraffin and 4 micron sections were taken for routine hematoxylin and eosin (H&E) stain and immunohistochemical (IHC) assay. IHC assay was performed by using a polymer-based colorimetric indirect immunoalkaline phosphatase method with a monoclonal antibody against the nucleoprotein of influenza A virus.

### HAI titer

Serum samples were treated with 3 volumes of receptor destroying enzyme (RDE) (Denka Seiken) at 37°C for 18 hours followed by inactivation of the RDE via incubation at 56°C for 30 min. Serum at a final dilution of 1:10 in PBS was added in duplicate to 96-well V-bottom plates, and further two-fold serial dilutions were generated. Four hemagglutination units of PR8 were added to each well and incubated for 30 min at RT. One-percent turkey RBCs (Lampire Biological Laboratories) in 50μL were added to each well and the plates were incubated for 30 min. HAI titers were determined as the highest dilution where RBCs movement was unrestricted by agglutination.

### Statistics

For differences in weight loss, the average percentage of original body weight of the B6 and Nox1*^/Y^ mice was compared by a Students *t* test for individual days p.i. Survival curves were compared using both the Log-Rank test and the Gehan-Breslow-Wilcoxon tests. HAI titers were compared for each of day 9 and day 15 data by Mann Whitney *t* test. Immune cell number or frequency, viral titers, and Bioplex data were compared by 2-way ANOVA with the Bonferonni multiple comparisons post-test, including data from both day 3 and day 6, or from both day 9 and day 15. Day 15 intracellular cytokine data were compared by 2-way ANOVA with the Bonferonni multiple comparison post-test. All error bars indicate standard deviations from the mean. All statistics were obtained using Prism (GraphPad).

After analysis of the data by 2-way ANOVA, we had situations where the ANOVA determined that there was a significant difference due to genotype between the two groups. This analysis takes all the data points into account, irrespective of day post-infection. These results are reported in the figure legend but not on the figures themselves. To further determine whether the significance between the two groups was in part due to a significant difference between the two genotypes at one particular day post-infection, we conducted post-tests. In some cases, the post-tests determined that there was a significant difference at one of the days post-infection. This was indicated on the figure with a star. In other cases, the post-tests could not determine a difference between the two genotypes at a specific day individually. This does not negate the significant difference between the two groups when all the data is taken into account. However, in these cases, we only described the difference by ANOVA in the figure legend without denoting it by any star symbol in the graphs.

## Supporting Information

S1 Fig Characterization of mice lacking catalytic domain of Nox1. *A* Schematic representation of exon 13 in wild type and Nox1*^/Y^ with FP, RP1, and RP2 showing forward and reverse primers used for genomic DNA PCR-based genotyping. *B*, PCR results from wild type and Nox1*^/Y^ showing 400bp and 200 bp PCR products respectively. *C*, beta-galactosidase staining in wild type and Nox1*^/Y^ are shown.
(TIF)

S2 Fig Lack of Nox1 catalytic activity does not affect the microscopic appearance of inflammation or dissemination of virus at day 3 or 6 p.i. *A*, Representative H&E staining on histological sections from mouse lungs taken at the indicated day p.i. Magnification, 100x. *B*, Representative NP-specific immunostaining (pink) on hematoxylin-stained histological sections from mouse lungs taken at the indicated d p.i. Magnification, 200x.
(TIF)
