# Supplementary figures and images for: NADPH Oxidase 1 Is Associated with Altered Host Survival and T Cell Phenotypes after Influenza A Virus Infection in Mice
Source: PLoS One. 2016 Feb 24;11(2):e0149864. doi: 10.1371/journal.pone.0149864 (PMC4766197; doi:10.1371/journal.pone.0149864)

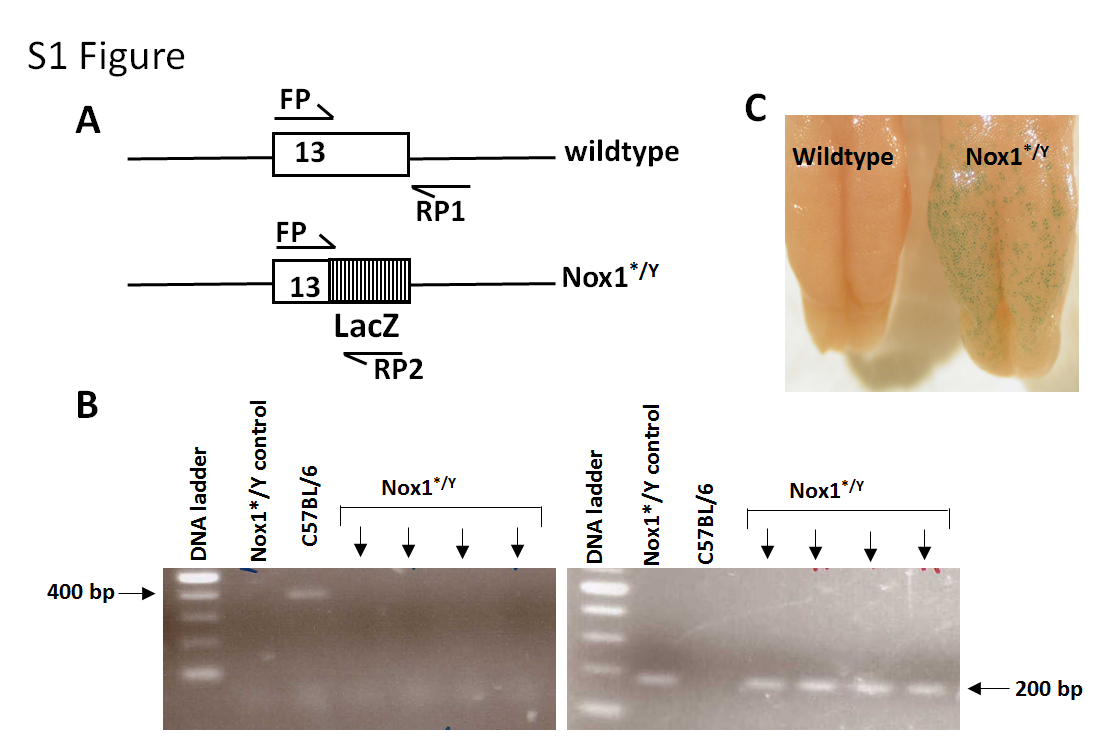

Supplement: S1 Fig — A Schematic representation of exon 13 in wild type and Nox1*/Y with FP, RP1, and RP2 showing forward and reverse primers used for genomic DNA PCR-based genotyping. B, PCR results from wild type and Nox1*/Y showing 400bp and 200 bp PCR products respectively. C, beta-galactosidase staining in wild type and Nox1*/Y are shown. (TIF) [file pone.0149864.s001.tif]

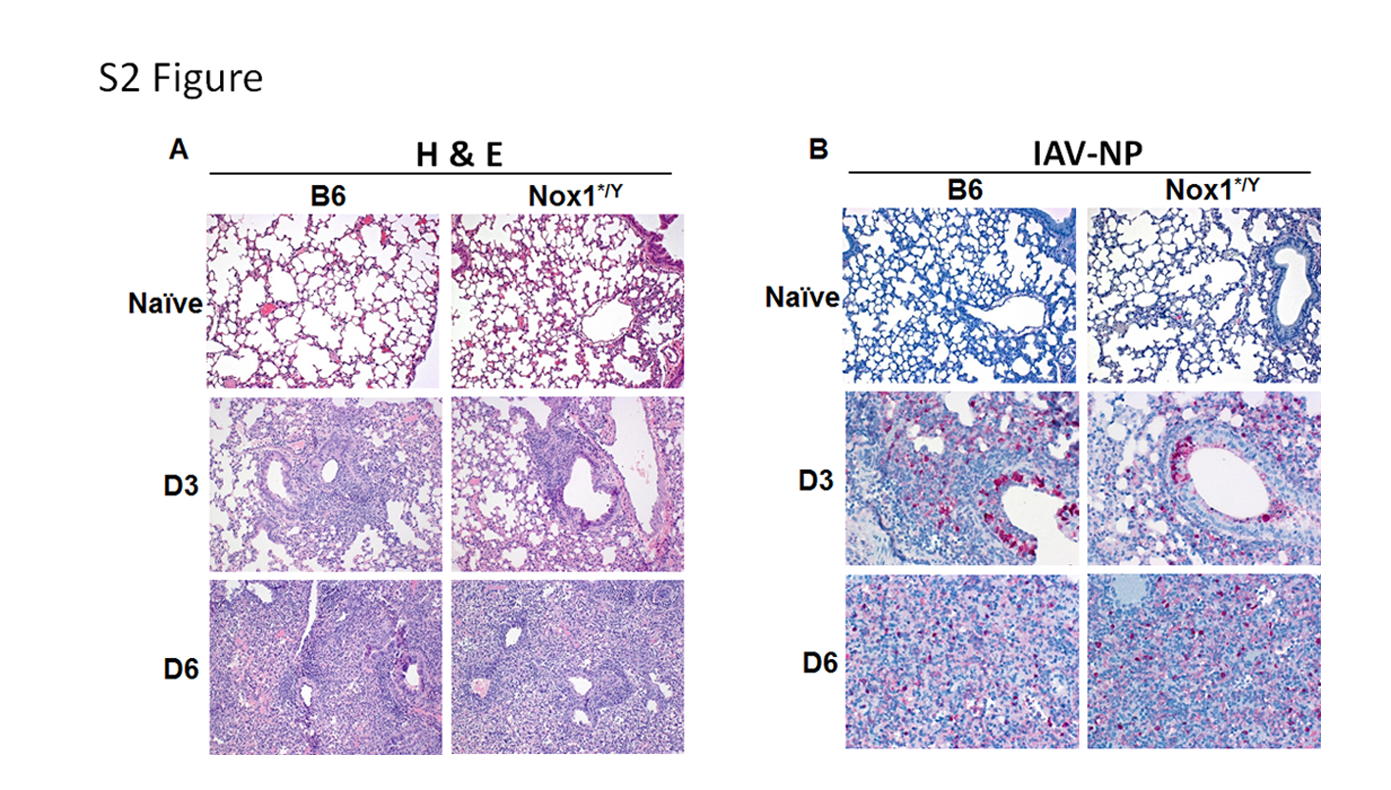

Supplement: S2 Fig — A, Representative H&E staining on histological sections from mouse lungs taken at the indicated day p.i. Magnification, 100x. B, Representative NP-specific immunostaining (pink) on hematoxylin-stained histological sections from mouse lungs taken at the indicated d p.i. Magnification, 200x. (TIF) [file pone.0149864.s002.tif]
